# General correlation between neonatal factors, primary and permanent tooth eruption and their interrelation in a population in german orthodontic practices

**DOI:** 10.1186/s12903-023-03153-1

**Published:** 2023-07-01

**Authors:** Stephan Christian Möhlhenrich, Volkan-Cem Korkmaz, Sachin Chhatwani, Gholamreza Danesh

**Affiliations:** Department of Orthodontics, University of Witten/Herdecke, Alfred-Herrhausen Str. 45, 58455 Witten, Germany

**Keywords:** Primary, Permanent, Tooth eruption, Time of birth, Birth weight, Birth height

## Abstract

**Background:**

The purpose of the study was to determine the possible relationship between the eruption of primary and permanent teeth and neonatal factors in German children according to gender.

**Methods:**

A cross-sectional survey study was performed in 10 German orthodontic practices. Using a questionnaire information about gender, time of birth (week of pregnancy), birth weight (g) and height (cm), and the age of first primary and first permanent tooth eruption (months/years) of 405 children (230 girls and 175 boys) were collected. A Mann–Whitney U-test was used for group comparisons, and correlations were verified using a Pearson test.

**Results:**

No correlation was found between neonatal factors (time of birth, birth weight, and birth height) and primary tooth eruption for male participants. However, for females a low correlation was found between the eruption of the first primary tooth and birth weight (r = -0.18, CI: -0.30 to -0.042, p = 0.011) and birth height (r = -0.19, CI: -0.32 to -0.054, p = 0.006). No correlations between neonatal factors and the eruption of the first permanent tooth were found for either gender. A moderate correlation was found between the first primary and first permanent tooth eruption (females: r = 0.30, CI: 0.16 to 0.43, p < 0.001; males: r = 0.22, CI: 0.059 to 0.35, p = 0.008).

**Conclusions:**

An earlier eruption of the primary teeth can be assumed with greater body weight and height at the time of birth for girls. For boys, the tendency is the opposite. However, there seems to be a catch-up growth effect due to the missing differences between both permanent tooth eruption times. Nevertheless, the first primary and the first permanent tooth eruption correlates in a German children population.

## Introduction

The timing and sequence of tooth eruption are important factors in orthodontic treatment planning and forensic dentistry to predict the age of a child. Tooth eruption is a continuous biological process in which a growing tooth erupts from the jaw through covered mucosa into the oral cavity and the fossa of the opposing tooth [1]. Different studies have demonstrated that the eruption of both dentitions is a sequential, systematic and age-specific process. In general, primary teeth appear between 6 and 8 months old, while permanent teeth erupt between the ages of 5 and 13 [2–5]. In this context, other variables, such as genetics, hormonal factors, geographic location, ethnicity, gender, economic status, nutrition and age, have an impact on when the teeth erupt [4–7]. Gender and neonatal-specific factors are the most tangible clinical parameters.

It has generally been found that girls usually get their permanent teeth earlier than boys [8, 9]. The greatest gender-specific difference observed concerns the canines of the maxilla, and the average retardation is 4 to 6 months. Typically, 97% of all girls have their first permanent molars by the age of 96 months, while the correlating age for boys is about 99 months. Similar results were reported by Kutesa et al. for African children from Uganda [10]. The earlier eruption in females can be explained by the generally earlier beginning of physical development.

Regarding the time of birth (week of pregnancy), the incidence of preterm birth varies from 5 to 13% in developed countries [11–14]. In this context, the systematic review by Almonaitiene et al. found that premature birth has a proven effect on the development of primary dentition [8]. Unfortunately, the data allow less distinct conclusions with regard to permanent dentition. However, the authors report that most studies found that the delayed eruption of both deciduous and permanent teeth is associated with preterm birth, but no differences in tooth development and eruption were identified between a preterm birth group and an age-corrected control group. Furthermore, the data suggest that the delayed eruption of deciduous teeth catches up as facial development continues [8].

Regarding height and weight, Kutesa et al. found a moderate correlation between these body characteristics and the dentition development of a population group in Uganda [10]. Similarly, Almonaitiene et al. reported a positive correlation between body size and weight and tooth eruption [8]. Taller and heavier children are slightly ahead in their dental development, whereas children who are inhibited in their overall development clearly show delayed dental development. Hilgers et al. reported that in overweight children, tooth eruption occurs on average 1.2 to 1.5 years before that of children with a normal body mass index [15]. Khan tested the relationship between height, weight, or body mass index and average tooth eruption times in Pakistani children in a cross-sectional study and found a statistically high positive correlation between height, weight, and socioeconomic factors, such as school education, and the eruption time of permanent teeth [16]. They concluded that the tooth eruption time of Pakistani children seems to be different in many aspects compared to children of other nationalities.

Only a few studies investigated the possible relationship between the tooth eruption of primary dentition and permanent dentition. In a cohort study, Poureslami et al. were able to confirm a significant correlation with regard to the eruption times of both kinds of teeth, but it was independent of socioeconomic status [17]. They showed that eruption of the first deciduous tooth delayed or earlier by 1 month was associated with an eruption of the first permanent tooth delayed or earlier by about 4 months on average.

The purpose of the present study is to investigate the possible relationship between primary and permanent tooth eruption, gender and neonatal factors in German children. It should be determined whether there is a systematic correlation between birth week, birth weight and birth height and the first primary or first permanent tooth eruption. Furthermore, it will be analyzed if there is a possible correlation between the beginning of the eruption of the deciduous and permanent dentition.

## Materials and methods

This cross-sectional study was performed in 10 randomly selected orthodontic practices in North Rhine-Westphalia, the most populous state in Germany, and Lower Saxony, the second largest state in Germany. The investigation was conducted between April and December 2016 as a survey. The ethical guidelines of the Helsinki Declaration and its later revisions were followed in all procedures carried out in this study, and ethical approval was given by the ethics committee of the University of Witten/Herdecke (189/2015).

The initial presentation to the orthodontic practices was made on the parents/guardians’ initiative or due consultative referral. An orthodontic treatment need was not a requirement for inclusion in the study. The children were selected according to Poureslami et al. [17] considering the following inclusion criteria: full-term healthy infants, born from uncomplicated pregnancies and deliveries; complete physical and mental health and no confounding medical history like having clefts or syndromes, agenesis or early tooth loss caused by caries or traumatic events and hormonal disturbances. The participants had to be between 4 and 15 years and born in Germany. Furthermore, each birth weight was included in the study.

Prior to the questioning, written consent was received from the parents/guardians on behalf of the children, and the children’s assent was obtained after participation was explained by a specially instructed member of each practice. The exclusion criteria were children with clefts or syndromes, agenesis or early tooth loss caused by caries or traumatic events and hormonal disturbances. The survey was carried out using a specially developed questionnaire, which the parents/guardians filled out. Participation in this survey was only agreed upon by the examiners if the parents/guardian were absolutely certain about the correctness of the provided information. The questionnaire included questions about:

- Date of birth and gender
- Time of birth (week of pregnancy)
- Birth weight (g) and height (cm)
- Age at the first primary tooth eruption (in months) and first permanent tooth eruption (in years).

### Statistical analysis

The a priori sample size calculation was performed using the G Power software (Heinrich-Heine-Universität, Düsseldorf, Germany) with the Wilcocon-Mann-Whitney test for mean differences between groups. Considering the data of Poureslami et al. [17] about the mean first tooth eruption time for primary (8.5 ± 3.2 months for boys and 6.9 ± 2.9 months for girls) and permanent tooth (87.9 ± 15.2 months for boys and 82.7 ± 15.6 month for girls) an effect size of 0.52 and 0.33 was determined respectively. Regarding the gender specific comparison, a necessary sample size of N = 85 resulted for the primary tooth eruption and of N = 197 for the permanent tooth eruption. A Shapiro–Wilk test revealed no normal distribution. Therefore, a nonparametric Mann–Whitney-U test was used for group comparisons in terms of gender. The correlations between the first primary or first permanent tooth eruption and the time of birth (week of pregnancy) and birth weight and height according to gender were verified by a Pearson test. The level of significance was set at *P* ≤ 0.05 by using the statistical program Prism (version 9; GraphPad Software Inc.). All results are expressed as mean ± standard deviation values, as well as minimum, median and maximum.

## Results

**Fig. 1:**
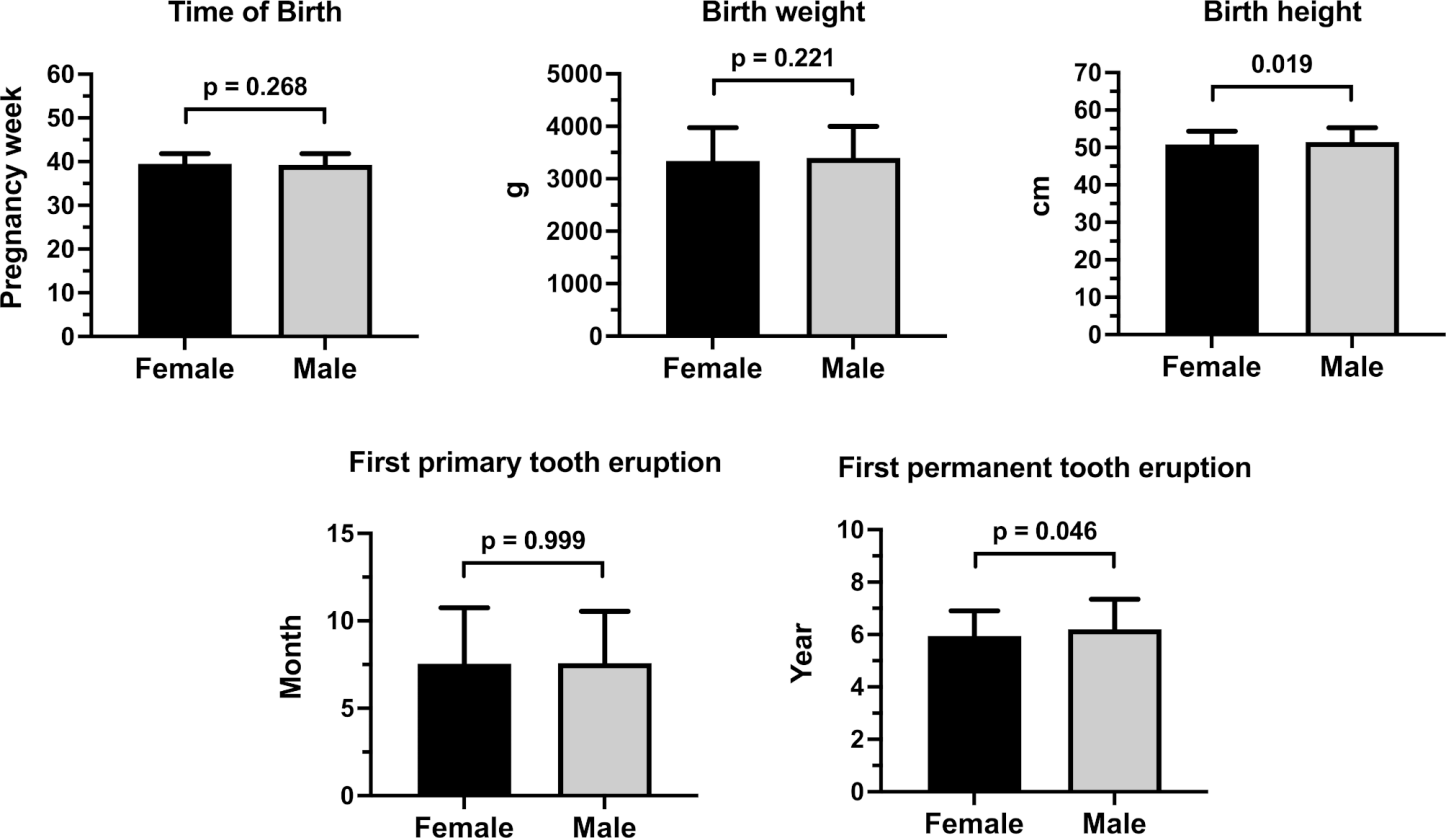
Bar chart of the mean values and standard deviations of the neonatal factors (time of birth, birth weight and birth height) and time of first primary and first permanent tooth eruption with corresponding p values of statistical comparison between the genders using a Mann–Whitney U test

**Fig. 2:**
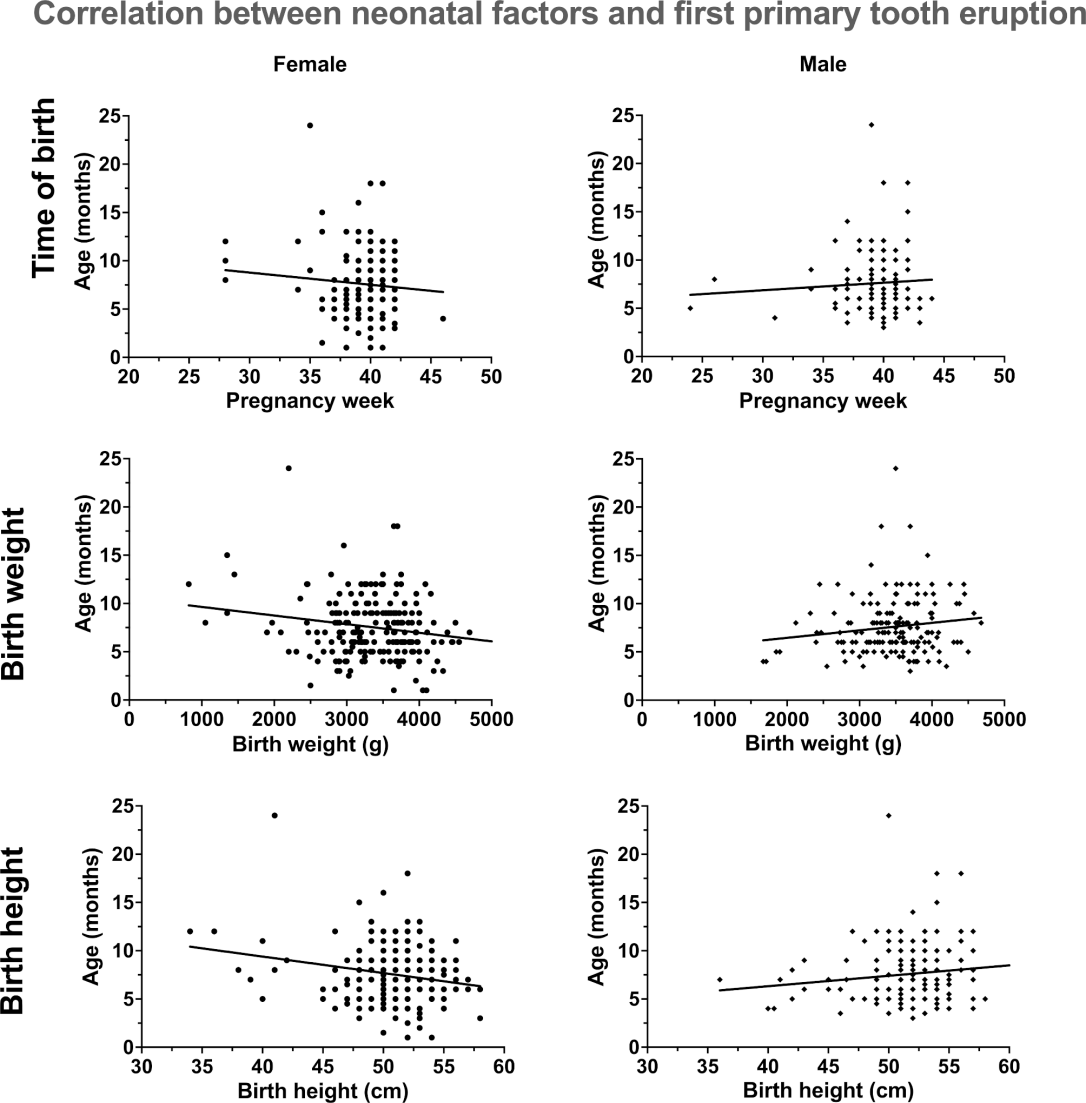
Scatter plots of possible correlation between neonatal factors (time of birth, birth weight and birth height) and first primary tooth eruption for both genders

**Fig. 3:**
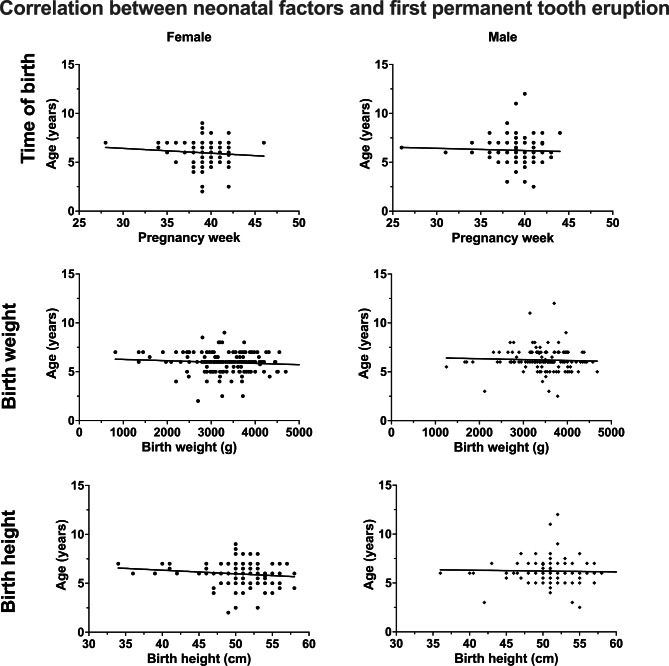
Scatter plots of possible correlation between neonatal factors (time of birth, birth weight and birth height) and first permanent tooth eruption for both genders

**Fig. 4:**
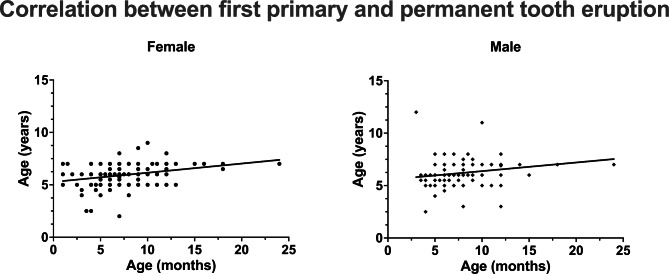
Scatter plots of possible correlation between first primary and first permanent tooth eruption for both genders

Table 1 describes the patient collective with regard to the physical constitution of the participants in terms of time of birth and time of first eruption of both dentitions. Figure 1 shows the corresponding boxplots and the corresponding *P*-values of the statistical comparisons between the genders for the neonatal factors and the eruption times of both dentitions based on a Mann–Whitney U test. Table 2 presents the results of the correlation analysis. Figure 2 shows the corresponding overall relationship of the first primary tooth eruption and the time of birth (week of pregnancy) and the birth weight and height according to gender. Figure 3 shows the same relationships for first permanent tooth eruption. Table 3; Fig. 4 demonstrate the correlation between the first primary and first permanent tooth eruption with regard to gender.

**Table 1:** Characterisation of the patient collective with regard to physical development at the time of birth and tooth eruption times of the first and second dentitions

Factors		N	Mean	SD	SEM	Min	Max
Week	Female	224	39.54	2.30	0.15	28	50
	Male	172	39.26	2.57	0.20	24	45
	Total	396	39.41	2.42	0.12	24	50
Weight (g)	Female	227	3339.00	633.80	42.07	820	5300
	Male	175	3399.00	598.50	45.25	1250	4680
	Total	402	3365.00	618.60	30.86	820	5300
Height (cm)	Female	224	50.82	3.52	0.24	34	58
	Male	173	51.48	3.77	0.29	36	63
	Total	397	51.11	3.65	0.18	34	63
Age at first deciduous tooth eruption (months)	Female	210	7.55	3.20	0.22	1	24
	Male	164	7.58	2.96	0.23	3	24
	Total	374	7.56	3.09	0.16	1	24
Age at first permanent tooth eruption (years)	Female	186	5.94	0.96	0.07	2	9
	Male	146	6.21	1.14	0.09	2.5	12
	Total	332	6.06	1.05	0.06	2	12

N: Numbers, SD: Standard deviation, SEM: Standard error of mean

**Table 2:** Correlations between the first primary or permanent tooth eruption and the time of birth (week of pregnancy) and birth weight and height depending on gender

Tooth eruption	Gender	Neonatal factor	Number of pairs	Pearson´s Rank Correlation (r)	95% confidence interval	P value
First primary	Female	Week	205	-0.087	-0.22 to 0.051	0.217
		Weight	210	-0.18	-0.30 to -0.042	0.011*
		Height	210	-0.19	-0.32 to -0.054	0.006*
	Male	Week	163	0.067	-0.087 to 0.22	0.390
		Weight	164	0.14	-0.011 to 0.29	0.069
		Height	164	0.14	-0.017 to 0.82	0.082
First permanent	Female	Week	182	-0.10	-0.24 to 0.044	0.172
		Weight	186	-0.092	-0.23 to 0.053	0.214
		Height	186	-0.13	-0.27 to 0.012	0.072
	Male	Week	145	-0.046	-0.21 to 0.12	0.581
		Weight	146	-0.045	-0.21 to 0.12	0.587
		Height	145	-0.028	-0.19 to 0.13	0.736

* Statistically significant different = p < 0.05

**Table 3:** Correlations between the first primary and permanent tooth eruption depending on gender

First tooth eruption	Birth parameters	Number of pairs	Pearson´s Rank Correlation (r)	95% confidence interval	P value
Primary vs. permanent dentition	Female	182	0.30	0.16 to 0.43	< 0.001*
	Male	142	0.22	0.059 to 0.37	0.008*

* Statistically significant different = p < 0.05

After completing the data collection, it was possible to access data sets from a total of 405 children—230 girls (56.5%) and 175 boys (43%). In some cases, the questionnaire was not completed in full (Table 1). The mean time of birth for the children was the 40th week of pregnancy. There were no statistical differences between the genders (female: 39.54 ± 2.30 weeks vs. male: 39.26 ± 2.57 weeks, p = 0.268). The patients had a mean birth weight of 3.37 ± 0.62 kg and a mean birth height of 51.11 ± 3.65 cm. Regarding birth weight, no difference between the genders was found (female: 3.34 ± 0.63 kg vs. male: 3.34 ± 0.60 kg, p = 0.221); however, there was some difference in terms of birth height (female: 50.82 ± 3.52 cm vs. male: 51.48 ± 3.77 cm, p = 0.019). The first dentition started at the age of 7.56 ± 3.09 months and the second at 6.06 ± 1.05 years. Concerning possible gender- related variances, no difference was found for the first tooth eruption (female: 7.55 ± 3.20 months vs. male: 7.58 ± 2.96 months, p = 0.999). However, for the second tooth eruption (female: 5.94 ± 0.96 years vs. male: 6.21 ± 1.14 years; p = 0.046) there was some difference.

Regarding the possible relationship between neonatal factors and the eruption of the first primary teeth, no correlation was found for any of the neonatal factors (time of birth, birth weight and birth height) for male patients. However, for females a low correlation was found between the first primary tooth eruption and birth weight (r = -0.18, CI: -0.30 to -0.042, p = 0.011) and birth height (r = -0.19, CI: -0.32 to -0.054, p = 0.006). No correlations between neonatal factors and the first permanent tooth eruption was found for female or male patients. However, a slight to moderate correlation was observed between the first primary and first permanent tooth eruption (females: r = 0.30, CI: 0.16 to 0.43, p < 0.001; males: r = 0.22, CI: 0.059 to 0.35, p = 0.008).

## Discussion

The overall aim of the present study was to investigate the existence and extent of systematic correlations between first tooth eruption in primary dentition and permanent dentition and whether these are possibly influenced by neonatal factors (gender, time of birth, birth weight and birth height). To that end, a survey study was conducted from April to December 2016 in 10 orthodontic practices in North Rhine-Westphalia and Lower Saxony, Germany.

There are some points in the study design that must be considered as critical and limited with regard to the interpretation of the present results and its associated meaning of significance. Concerning the participant selection, it must be noted that other potential factors, such as genetic, nutritional or hormonal factors, were not taken into account or excluded, which must be considered as drawback due their effect have already described [8]. The same is actual for craniofacial or systemic diseases. In addition, a bias must be assumed due the preselection of the participants by performing the survey in orthodontic practices. These children may already have a finding requiring orthodontic treatment need in tooth eruption disorders, like lack of space. Unfortunately, an actual need for treatment was not documented in this the survey. As a consequence, the present study results are only representative for a limited population. In addition, the gender distribution also deviates slightly from the regular distribution, as girls presented comparatively more frequently to the orthodontic practices (girls: 56.8% vs. boys: 43.7%). In addition, it must be critically seen that the participants answered the questionnaire retrospectively. This is certainly possible for the influencing factors (neonatal factors), but probably not exactly for the outcome variables (primary and permanent tooth eruption), even if only children were included for whom parents/guardians were absolutely certain about the correctness of the provided information. Statements by the parents regarding the change of teeth, which frequently occurred years ago, is potential error sensitive and thus only scientifically valid to a limited extent. On the other hand, higher scientific evidence can only be achieved by prospective data collection in terms of a monitoring study, which is complex to perform and difficult to realize if a similar same size should be reached. In this context, another discussible aspect is the statistical power of the present survey. Although an appropriate sample size calculation was carried out in advance of the study, a deviation from the expected normal distribution became apparent after the survey was carried out. Thus, non-parametric tests were used for the group comparisons. But in retrospect, a larger sample size would have been more helpful. Within these aspects, the evaluation and interpretation of the present results must be critically. The determined effects are comparatively small, which is also due to the limitations mentioned above.

### Relationship between birth week, birth weight and birth height and first primary tooth eruption

The significance of birth week and birth size in relation to the timing of the first deciduous tooth eruption has been debated in the literature. Based on international comparisons, regional differences seem to exist. In this context, Soliman et al.’s study of 1132 Egyptian children (565 girls, 567 boys) showed that boys seem to be ahead of girls with regard to the number of erupted teeth in relation to birth weight and height [3]. In their study of 1601 children in India aged between 4 and 36 months, Verma et al. reported a systematic influence in terms of birth height and weight, and the authors noted a delay compared with children from other countries [18]. In a longitudinal study of 40 Brazilian children born prematurely and with a birth weight of less than 1500 g, Neto and Falcão found eruption of the first deciduous tooth occurred at about 11.0 ± 2.1 months [19]. However, with regard to the subsequently statistically corrected age of 9.6 ± 1.9 months, the authors concluded that no relationship exists between nutritional status at birth and first tooth eruption. In contrast, Wang et al. postulated that for Chinese preterm infants age is indeed a factor influencing the time of eruption [20]. Based on a collective of 2230 children aged 3 to 36 months, 89 of them preterm, it was found that the time of first primary tooth eruption was significantly later in preterm infants (8.4 vs. 7.3 months). Aktoren et al. confirmed an apparent delay in deciduous tooth eruption in preterm infants [21]. Each study supports the statement that the week of birth has an effect on the eruption time of the first deciduous tooth. Regarding weight, Un Lam et al. found that the early eruption of first primary teeth was associated with childhood obesity and diabetes mellitus [22]. In a study of 1033 children, they determined that early tooth eruption was significantly associated with a child’s weight gain rate in the first 3 months of life.

In the present investigation, no significant correlation between birth week and age at first primary tooth eruption was detectable. However, regarding birth weight and age at eruption of the first deciduous tooth, a significant correlation was found for the female participants. The higher the birth weight of the girls, the younger they were at the first primary tooth eruption. The opposite trend was observed in boys; the higher the birth weight, the older the male participants were when the first deciduous tooth erupted. However, for boys this was not confirmed statistically. The analysis of the present data to determine a correlation between birth height and age at first primary tooth eruption also showed a significant correlation for the female participants. Similarly, the effects were opposite in boys and girls, although they were not statistically significant for the male participants. This means that for girls, the larger they were at birth, the younger they were at the eruption of the first deciduous tooth and for boys, the larger they were at birth, the later the first primary tooth erupted.

Therefore, our results confirm those studies that do not describe a correlation between birth week and the first primary tooth eruption but show that there seems to be gender-specific correlations with regard to birth size or weight. This suggests that, compared to boys, girls who are comparatively heavier and taller at birth may have earlier eruption of deciduous teeth.

### Relationship between birth week, birth weight and birth height and first permanent tooth eruption

Concerning gender, there is agreement in the literature that girls’ permanent teeth erupt earlier than boys’ permanent teeth [23–27]. In general, the difference between gender-specific eruption times is about 4 to 6 months, particularly with regard to the second canines. The effect of preterm birth on the development and eruption of permanent teeth has been examined in various studies [28–30]. Backstrom et al. found no differences between a group of preterm children and a control group [30]. Harila-Kaera et al. reported an earlier eruption of permanent first molars and incisors in premature black and white children compared to controls [31]. The authors suggested that various postnatal factors and an accelerated growth period (catch-up effect) with associated unspecified factors could affect the eruption of permanent teeth. Previous studies have reported a positive relationship between birth height and weight and the eruption of teeth [6, 32]. Basically, in taller and heavier children first permanent teeth emerge earlier, while retarded physical growth is significantly related to delayed tooth eruption. Furthermore, Hilgers et al. found a positive relationship between obesity in children and dental development [15]. They found that teeth tend to erupt on average 1.2 to 1.5 years earlier in overweight children compared to children with a normal body mass index.

In contrast to these findings, in the present study no significant correlation between week of birth, birth weight or height and the age at first permanent tooth eruption was found, either in the overall collective or in girls or boys. Therefore, previous interdependencies concerning the second dentition cannot be confirmed.

### Relationship first primary and permanent tooth eruption

Associations have been reported between maturity of primary dentition and permanent dentition showing that a small number of primary teeth at 1 year of chronological age is related to less mature permanent teeth at an age between 9 and 11 years in preterm children [30]. Poureslami et al. considered socioeconomic status (SES) in investigating the relationship between the first deciduous and first permanent tooth eruption and the variation in eruption time in a 9-year population-based cohort study. Three hundred and seven children were examined at bimonthly intervals during the first and second years of life and then at 6-month intervals until the eruption of first permanent teeth, and the eruption times of all teeth were recorded for each participant. The authors found a direct strong correlation between the eruption time of the first primary teeth and that of the first permanent teeth. They reported that a 1-month delay or the early eruption of the first deciduous tooth resulted in a 4.21-month delay or the early eruption of the first permanent tooth. However, no significant correlation was observed concerning SES regardless of the type of dentition. In this context, the present results confirm these findings. A statistically significant correlation was found between the first primary and first permanent tooth eruption, both for the total collective and for the genders separately, which means the higher the age at the first primary tooth eruption, the higher the age at the first permanent tooth eruption.

## Conclusion

No systematic correlation between the week of birth and the time of the first primary or first permanent tooth eruption was found. However, it is determined for girls that with increasing body weight and size at the time of birth, an earlier eruption of the primary teeth has to be assumed. For boys, the tendency is the opposite. Regarding the second dentition, there is no such correlation. This is probably due to the catch-up growth effect, which has already been discussed in the literature. But there is a systematic, statistically significant correlation between first primary tooth eruption and first permanent tooth eruption. However, the present findings are limited and must be critical considered with regard to the low statistical significance, which is attributed to the small sample size, as well as the underlying low validity, which based on the character of the present participant survey. This is attributed to the small sample size and the participant related reliability about the retrospective collected measurements. In order to improve this, prospective epidemiological research like monitoring studies will be necessary, which could then also analyse the entire dentition.

## Data Availability

The datasets used and/or analysed during the current study are available from the corresponding author upon reasonable request.
